# Linking Cancer Cachexia-Induced Anabolic Resistance to Skeletal Muscle Oxidative Metabolism

**DOI:** 10.1155/2017/8018197

**Published:** 2017-12-11

**Authors:** Justin P. Hardee, Ryan N. Montalvo, James A. Carson

**Affiliations:** ^1^Department of Exercise Science, University of South Carolina, Rm. 405 Public Health Research Center, 921 Assembly Street, Columbia, SC 29208, USA; ^2^Center for Colon Cancer Research, University of South Carolina, Rm. 614 Jones PSC Bldg, 712 Main Street, Columbia, SC 29208, USA

## Abstract

Cancer cachexia, a wasting syndrome characterized by skeletal muscle depletion, contributes to increased patient morbidity and mortality. While the intricate balance between protein synthesis and breakdown regulates skeletal muscle mass, the suppression of basal protein synthesis may not account for the severe wasting induced by cancer. Therefore, recent research has shifted to the regulation of “anabolic resistance,” which is the impaired ability of nutrition and exercise to stimulate protein synthesis. Emerging evidence suggests that oxidative metabolism can regulate both basal and induced muscle protein synthesis. While disrupted protein turnover and oxidative metabolism in cachectic muscle have been examined independently, evidence suggests a linkage between these processes for the regulation of cancer-induced wasting. The primary objective of this review is to highlight the connection between dysfunctional oxidative metabolism and cancer-induced anabolic resistance in skeletal muscle. First, we review oxidative metabolism regulation of muscle protein synthesis. Second, we describe cancer-induced alterations in the response to an anabolic stimulus. Finally, we review a role for exercise to inhibit cancer-induced anabolic suppression and mitochondrial dysfunction.

## 1. Introduction

Cachexia, a complex wasting syndrome characterized by skeletal muscle mass depletion, is prevalent in many cancers types and cannot be reversed by standard nutritional treatment [1, 2]. Approximately 20–40% of all cancer-related deaths have been attributed to cachexia, and skeletal muscle mass loss is directly associated with cancer patient morbidity and mortality [3, 4]. Skeletal muscle depletion reduces chemotherapy adherence, increases susceptibility to treatment toxicity, decreases physical function, and impairs psychosocial ability [1, 5–7]. Given the importance of muscle mass for systemic metabolic health and survival, an improved understanding of the mechanisms associated with cachexia development and progression should serve to enhance cancer patient treatment.

Skeletal muscle responds to both systemic and local environmental stimuli. Inflammation, insulin resistance, and hypogonadism are systemic perturbations associated with cancer that can disrupt skeletal muscle energy metabolism and proteostasis during cancer cachexia [8, 9]. While cancer-induced suppression of basal protein synthesis has been reported in patients and preclinical cachexia models [10–12], this suppression does not appear to fully account for the severe muscle wasting observed with cachexia. Furthermore, the importance of the cyclic anabolic stimulation of protein synthesis by nutrients and exercise has been firmly established [13]. Conversely, the inability to stimulate protein synthesis has been termed “anabolic resistance” and has an established role in the regulation of muscle mass loss with aging [13, 14]. While our mechanistic understanding of cachexia-induced protein synthesis suppression, protein degradation activation, and disrupted oxidative metabolism in skeletal muscle continues to develop [8, 15, 16], we still have a limited understanding of how these processes are interconnected for regulating the skeletal muscle response to the cancer environment.

There is considerable evidence that a muscle's capacity for oxidative metabolism can influence wasting susceptibility [8, 17]. Furthermore, muscle oxidative metabolism and protein turnover are disrupted in cachectic muscle [8]. While these two critical processes have historically been examined independently during skeletal muscle wasting, recent evidence has started to establish a linkage between these processes in the regulation of cancer-induced muscle wasting. Therefore, the primary objective of this review is to emphasize dysfunctional oxidative metabolism's connection with anabolic resistance during cancer-induced muscle wasting. First, we review oxidative metabolism regulation of skeletal muscle protein synthesis. Then, we describe cancer-induced alterations in the response to an anabolic stimulus. Finally, we review a role for exercise to inhibit cancer-induced anabolic suppression and mitochondrial dysfunction.

## 2. Muscle Oxidative Metabolism Regulation of Protein Synthesis

Skeletal muscle's metabolic capacity provides the basis for many functions that are critical for health and quality of life. Muscle fiber types have been linked to both contractile and metabolic properties [18], which are highly plastic and can be influenced by the systemic and local microenvironment. Classically, fiber types are classified by myosin heavy chain isoform expression (e.g., “slow” type I and the “fast” types IIA, IIX, and IIB), which can differ substantially in mitochondrial content and metabolic enzyme capacities [18, 19]. Generally, the rate and capacity for protein synthesis are greater in oxidative muscles when compared to glycolytic muscles, which has been related to total RNA capacity and a higher rate of protein turnover in oxidative muscles [20]. Additionally, rates of protein synthesis demonstrate fiber type differences in rodent and human skeletal muscle [21, 22]. While a causal relationship exists between oxidative phenotype and protein turnover, the disruption of oxidative metabolism can negatively impact both basal and induced protein synthesis in muscle. The following section highlights the current understanding of muscle oxidative metabolism regulation of basal protein synthesis.

### 2.1. Overview of Basal Protein Synthesis Regulation

Translational efficiency (polypeptide synthesis per ribosome) and capacity (total number of ribosomes) serve as critical control points for muscle protein synthesis. Translation efficiency is regulated at initiation, elongation, and termination steps [23]. Significant advances have been made in our understanding of the mechanisms regulating initiation in skeletal muscle. In brief, translation initiation includes (1) binding of the GTP-bound form of eukaryotic initiation factor 2 (eIF2) and initiator methionyl-tRNA (met-tRNA_i_) complex to the small (40S) ribosomal subunit to form the 43S preinitiation complex (PIC), (2) binding of the 43S PIC to the 7-methylguanosine (m^7^G) cap of the 5′ end of messenger RNA (mRNA), and (3) 43S PIC scanning of the mRNA for an AUG initiation codon [24, 25]. Upon AUG recognition, the large (60S) ribosomal subunit joins the 40S subunit to form an 80S initiation complex capable of promoting elongation [25]. Several eukaryotic initiation factors (e.g., eIF2, eIF3, and eIF5) and binding proteins (e.g., poly(A)-binding protein (PABP)) have critical roles in each one of these processes and can be regulated by cellular environments such as stress or nutrient availability [23]. Indeed, eIF2 activity is thought to be the rate-limiting step for translation initiation [24, 26]. The phosphorylation of eIF2*α* on Ser51 can prevent the formation of the eIF2•GTP•Met-tRNAi complex and inhibit global protein synthesis through competitive inhibition of the 5-subunit guanine nucleotide exchange factor (GEF), eIF2B [26]. Several nutrient and energy sensitivity kinases such as general control nonderepressible 2 (GCN2), protein kinase RNA- (PKR-) activated, and protein kinase R- (PKR-) like endoplasmic reticulum kinase (PERK) regulate eIF2*α* phosphorylation in response to atrophic stimuli [27]. Collectively, translation initiation regulation has emerged as a highly dynamic and coordinated process that is tightly linked to nutrient availability and energy stress.

**Figure 1:**
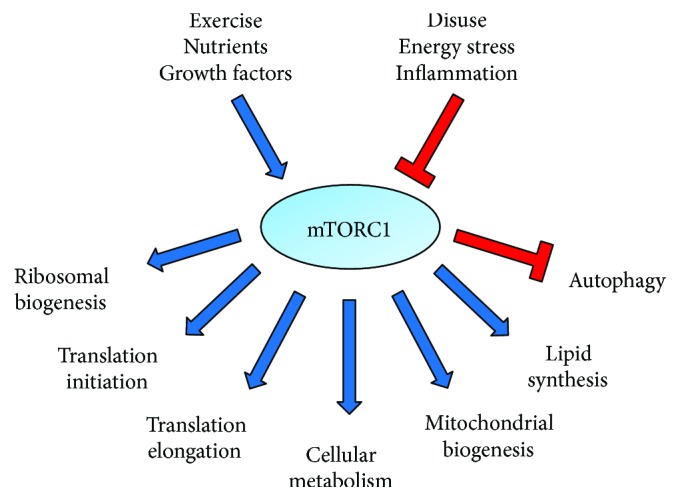
mTORC1-regulated cellular processes. mTORC1 has been shown to stimulate cellular processes related to protein synthesis, cellular metabolism, mitochondrial biogenesis and function, and lipid synthesis. mTORC1 has also been described in the inhibition of autophagy. Positive regulators of mTORC1 signaling include exercise, nutrients (e.g., amino acids), and growth factors (e.g., insulin and insulin-like growth factor-1). Negative regulators of mTORC1 signaling include disuse, energy stress (e.g., nutrient deprivation and unfolded protein response), and inflammation.

**Figure 2:**
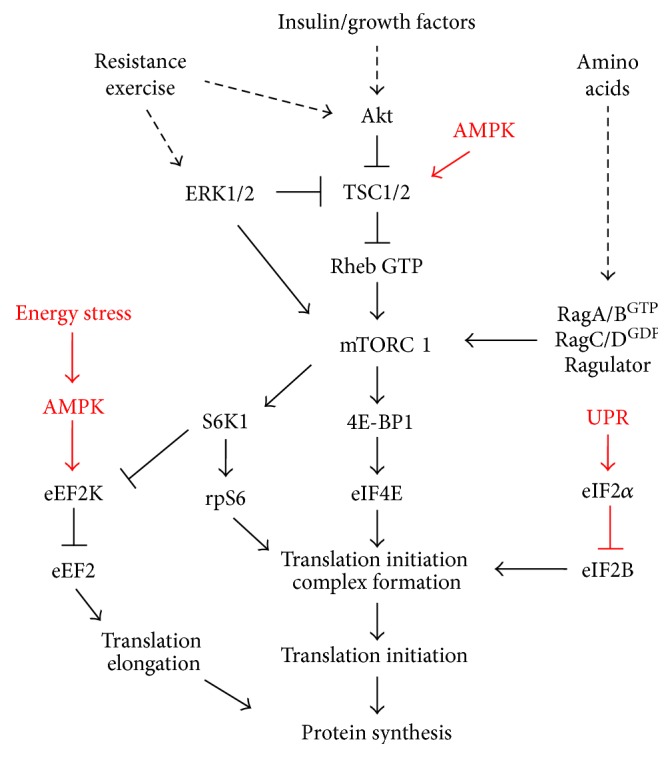
mTORC1 signaling regulation by acute anabolic stimuli. Exercise, growth factors, and nutrients can positively regulate muscle mTORC1 signaling. Cancer cachexia can suppress mTORC1 signaling through disrupted oxidative capacity and mitochondrial quality control. Disrupted muscle homeostasis can promote energy stress and an unfolded protein response, which negatively regulate mTORC1 signaling at several levels. Black lines: positive regulation. Red lines: negative regulation. ERK, extracellular signal-related kinases; Akt, protein kinase B; TSC1/2, tuberous sclerosis complex 1 and 2; Rheb, ras homolog enriched in brain; mTORC1, mammalian target of rapamycin complex 1; RagA/B, RagA and RagB heterodimer; RagC/D, RagC and RagD heterodimer; AMPK, AMP-activated protein kinase; S6K1, ribosomal protein S6 kinase beta-1; S6, ribosomal protein S6; 4E-BP1, eukaryotic translation initiation factor binding protein 1; eIF4E, eukaryotic initiation factor 4E; eEF2K, eukaryotic elongation factor 2 kinase; eEF2, eukaryotic elongation factor 2; eIF2*α*, eukaryotic initiation factor alpha; eIF2B, eukaryotic initiation factor 2B; UPR, unfolded protein response.

The mammalian target of rapamycin (mTOR) is an established regulator of cellular processes related to metabolism and growth (Figure 1). mTOR is a serine/threonine kinase that interacts with several proteins to form two distinct complexes, mTOR complex 1 (mTORC1) and 2 (mTORC2). mTORC1 has been investigated most widely for its role in the regulation of processes related to protein synthesis through the control of translational efficiency and capacity in muscle. In contrast, mTORC2 has been implicated in cell survival, cytoskeletal organization, and metabolism [28]. mTORC1 serves as a critical integration point for anabolic stimuli such as growth factors, nutrients, and mechanical load (Figure 2) [28, 29]. Upon activation, mTORC1 directly phosphorylates the eukaryotic translation initiation factor 4E (eIF4E) binding protein 1 (4E-BP1) and S6 kinase 1 (S6K1) to promote protein synthesis [30]. The hyperphosphorylation of 4E-BP1 prevents binding to eIF4E and the formation of 4E-BP1-eIF4E complex, resulting in the assembly of the eIF4F complex and translation initiation. The activation of mTORC1 has also been implicated in cap-dependent translation, translation elongation, and ribosomal biogenesis. Indeed, mTORC1 signaling has been implicated in the inhibition of eukaryotic elongation factor 2 kinase (eEF2K) activity [31]. The eukaryotic elongation factor 2 (eEF2) is responsible for the translocation of peptidyl-tRNA at the ribosome during translation, and eEF2 phosphorylation by eEF2K inhibits its activity [32]. Related to ribosomal capacity, mTORC1 can regulate translational machinery components by promoting synthesis of mRNAs encoding ribosomal proteins (RPs) and the transcription of ribosomal RNAs (rRNAs) [28, 33]. Thus, mTORC1 signaling serves as a critical regulatory point for growth and metabolic alterations that could be targeted in muscle wasting conditions.

### 2.2. Role of Oxidative Metabolism in Protein Synthesis and mTORC1 Signaling

Mitochondrial respiration and ATP synthesis are intimately linked to cellular energy utilization, and thus mitochondria exert control over ATP-utilizing processes [34]. Therefore, it is not surprising that mitochondrial function has an established role in the regulation of cellular processes related to protein synthesis and growth [35]. Protein synthesis is an energy-demanding process that accounts for approximately 20–30% of mammalian ATP consumption [34, 36]. While specific synthesis rates can vary between muscle fractions (e.g., myofibrillar, sarcoplasmic, and mitochondria) [37], the energy required for protein metabolism has been linked to de novo protein synthesis. Moreover, many nonribosomal energy-consuming enzymes such as ATP-dependent RNA helicases, AAA-ATPases, GTPases, and kinases are required for the synthesis, assembly, and maintenance of ribosomes [38, 39]. Collectively, the overall energetic cost of protein synthesis implicates a role for mitochondrial integrity and function in this important anabolic process.

The energy-sensing molecule 5′-AMP-activated protein kinase (AMPK) regulates energy homeostasis in response to metabolic stress [40] and is well suited to couple cellular energy status to protein synthesis. AMPK is a highly conserved heterotrimeric kinase complex composed of a catalytic *α*-subunit (*α*1 and *α*2) and two regulatory (*β*- and *γ*-) subunits [41]. The *β* subunit contains a glycogen-binding domain (GBD), whereas the *γ* subunit is responsible for nucleotide binding [42]. Several upstream kinases have been identified that regulate AMPK activity: the serine-threonine liver kinase B1 (LKB1) and calcium/calmodulin kinase kinase-*β* (CaMKK*β*) [41]. AMPK is activated under conditions of energy stress when intracellular ATP levels decline and intracellular AMP increases (high intracellular AMP/ATP ratio). AMPK activation stimulates ATP synthesis while concurrently inhibiting ATP consumption [43]. Thus, AMPK has been implicated in the regulation of mTORC1 [44], ubiquitin E3 ligase expression [45], autophagy/mitophagy [46, 47], and mitochondrial biogenesis [48]. AMPK inhibits mTORC1 signaling through the direct phosphorylation of TSC2 and Raptor [49–51]. TSC2 phosphorylation inhibits mTORC1 signaling through the maintenance of Ras homologue enriched in brain (Rheb) in its GDP-bound inactive state [50, 52]. Moreover, Raptor phosphorylation at two highly conserved serine residues (S722 and S792) can also suppress mTORC1 activity [51]. Thus, AMPK activation in response to mitochondrial dysfunction could serve to suppress mTORC1 activation of protein synthesis.

### 2.3. Mitochondrial Dysfunction and Energetic Stress

Mitochondrial dysfunction impairs the ability to convert nutrients (carbohydrate, fatty acids, etc.) into energy while concomitantly producing reactive oxygen species (ROS) that can inhibit biosynthetic pathways required for metabolic homeostasis [53]. Moreover, oxidative stress-induced accumulation of damaged and misfolded proteins can cause endoplasmic reticulum (ER) stress and an unfolded protein response (UPR). Thus, global protein synthesis is downregulated to resolve dysfunctional protein accumulation. The following sections highlight signaling pathways that link disrupted mitochondrial function to suppressed protein synthesis.

#### 2.3.1. ROS

Subsarcolemma and intermyofibrillar mitochondria are prominent sources of skeletal muscle ROS generation [54–56]. While ROS can serve as signaling molecules to regulate cellular processes [57], excessive ROS production can lead to the oxidation of proteins, lipids, and DNA [58]. The relative contribution of ROS to induce oxidative stress and damage is dependent on both ROS production and the level of cellular antioxidants [55]. For example, glycolytic fibers have increased susceptibility to oxidative stress, which involves enhanced ROS generation and decreased scavenging capabilities, compared to oxidative fibers [59]. Therefore, the cycle of aberrant ROS production and damage accumulation has been attributed to muscle atrophy associated with aging and inflammatory pathologies.

#### 2.3.2. ER Stress and UPR

Pathological stressors such as dysregulated protein synthesis, accumulation of misfolded proteins, calcium imbalance, and energy deprivation can all induce ER stress [60]. Excessive ROS production can induce ER stress and activate the UPR [61]. Additionally, the UPR can be activated when the balance between newly synthesized peptides and degrading misfolded proteins occurs, and an exacerbated UPR can disrupt muscle homeostasis. Under normal conditions, the UPR functions to return homeostasis through three major pathways: inositol-requiring protein (IRE1), RNA-activated protein kinase-like endoplasmic reticulum kinase (PERK), and activating transcription factor-6 (ATF6) [61–63]. PERK promotes protein synthesis inhibition through the inactivation of eIF2*α* [64]. ATF6 in conjunction with ATF4 and XBP1 can decrease ROS and ER stress [65]. IRE1 has both kinase and endoribonuclease activities to alter gene expression favoring apoptosis signaling or metabolic adaptations that promote survival [61, 64]. Overall, ER stress and UPR activation inhibit translation at the level of initiation through the phosphorylation of eIF2*α*, thereby preventing further unfolded protein accumulation, while preserving nutrients and energy [60]. Thus, while ER stress and UPR are adaptive responses to maintain homeostasis, the aberrant activation of these cellular processes can compromise muscle mass and metabolic function.

#### 2.3.3. Insulin Resistance

Insulin resistance accompanies many wasting conditions [66], and disrupted oxidative metabolism has been implicated in decreased skeletal muscle insulin sensitivity [67]. Altered mitochondrial function and metabolic flexibility can disrupt glucose and lipid homeostasis, which are also associated with insulin resistance [68, 69]. Disrupted muscle glycolytic flux is associated with altered amino acid utilization as tricarboxylic acid (TCA) intermediates [70], which could negatively affect protein metabolism. Indeed, the TCA cycle has emerged as a critical point in metabolic regulation linking bioenergetics with anabolic and catabolic pathways. Lipid accumulation and bioactive lipid intermediates, such as diacylglycerols (DAG) and ceramides, can inhibit insulin signaling and contribute to anabolic resistance. Increased ceramide can inhibit Akt through the activation protein kinase C (PKC) isoforms or protein phosphatase 2A (PP2A) [71]. Moreover, ceramide can impair insulin receptor function through glycosphingolipid GM3 synthesis [72, 73]. DAG can also inhibit insulin signaling transduction through PKC activation and serine phosphorylation of insulin receptor substrate 1 (IRS-1) [74, 75]. Lastly, ceramide can modulate nutrient uptake through the regulation of the sodium-dependent neutral amino acid transporter 2 (SNAT2) [76, 77], which could impair amino acid activation of mTORC1. Collectively, impaired insulin sensitivity through altered oxidative metabolism could have significant ramifications on metabolic flexibility during pathological conditions.

### 2.4. Reciprocal Regulation through mTORC1

Several lines of evidence suggest that mTORC1 signaling can regulate energy production and oxidative metabolism across various cell types [78–80]. mTORC1 activity and mTOR-Raptor complex formation has been tightly correlated with mitochondrial metabolism [80]. Specifically, lower mTORC1 activity is associated with decreased mitochondrial oxygen consumption, whereas increased mTORC1 activity (e.g., TSC2 knockdown) is accompanied by enhanced mitochondrial oxygen consumption [80]. Moreover, the inhibition of mTORC1 signaling reduces mitochondrial respiration (coupled and uncoupled), impairs TCA cycle activity, and lowers ATP production capacity [81]. In line with a potential regulatory role of mTORC1 activity on oxidative metabolism, reduced mitochondrial content and function has been observed in muscle-specific mTOR or raptor knockout (RAmKO) mice [82, 83]. Interestingly, mTORC1 inactivation in these mice is associated with altered systemic metabolic homeostasis and muscle atrophy/dystrophic phenotype [82, 83]. mTORC1 signaling also has the potential to perturb mitochondrial function through the regulation of translation and transcription to modulate expression of genes involved in oxidative metabolism [78, 81, 84]. The activation of mTORC1/4E-BP pathway has been implicated in the control of energy homeostasis via the translation of nuclear-encoded mitochondria-related genes such as mitochondrial transcription factor A (TFAM), mitochondrial ribosomal proteins, and complex I and IV components [81]. mTORC1 can also regulate mitochondrial content and function by modulating transcription factors that regulate energy metabolism [78, 85]. Indeed, mTORC1 has been implicated in the regulation of mitochondrial gene expression through the direct modulation of Yin Yang 1 (YY1) and PGC-1*α* transcriptional complex activity [78]. In skeletal muscle, YY1 physically interacts with and recruits PGC-1*α* to the promoters of nuclear mitochondrial genes, and the inhibition of mTORC1 by rapamycin prevents the coactivation of YY1 by PGC-1*α* [78]. In addition to its role in translation and transcription, mTORC1 has also been implicated in the regulation of autophagy, a process associated with catabolism and the regulation of mitochondrial quality control. mTORC1 inhibits autophagy by phosphorylating the proautophagic kinase ULK1, which prevents its association and subsequent activation by AMPK [46, 86]. Thus, chronic mTORC1 suppression could accelerate autophagy and perturb mitochondrial homeostasis. Collectively, these studies highlight a critical role for mTORC1 signaling in energy homeostasis through the regulation of protein synthesis and oxidative metabolism. Future research is warranted to determine the complex relationship between suppressed mTORC1 signaling and disrupted oxidative metabolism during pathological conditions.

### 2.5. Cancer-Induced Changes in Protein Synthesis and Oxidative Metabolism

Disrupted protein synthesis and oxidative metabolism have established roles in many wasting conditions and are intricately linked to wasting processes during cancer cachexia progression. Several studies have demonstrated suppressed basal protein synthesis in preclinical models of cancer cachexia [10, 87–89]. Interestingly, muscle protein synthesis is suppressed during the initial stages of weight loss (<5% body weight loss) and is further reduced throughout cachexia progression in tumor bearing mice [11]. Cancer cachexia has the potential to disrupt protein translation at several regulatory steps (e.g., initiation, elongation, and termination), and the regulation of translation initiation during cancer cachexia has been an active area of investigation. The phosphorylation of eIF2*α*, which attenuates translation initiation and overall protein synthesis, is elevated in cancer patients and preclinical models [90–92]. Moreover, mTORC1 signaling is suppressed during late stage cachexia [11, 93, 94], which corresponds to disrupted S6K1 and 4EBP-1 regulation [11, 91]. Lewis lung carcinoma (LLC) conditioned media or recombinant IL-6 can suppress mTORC1 signaling in C_2_C_12_ myotubes [94–96], which highlights a direct role of circulating factors on muscle. Elevated eEF2 phosphorylation has also been observed in cachectic mice bearing Murine adenocarcinoma 16 (MAC16) tumors [91]. Collectively, these studies highlight a role for translation initiation and elongation in disrupted protein synthesis regulation, which may serve as a potential therapeutic target to treat or prevent cancer cachexia.

Disrupted muscle oxidative metabolism coincides with suppressed protein synthesis and mTORC1 signaling [97, 98]. Recent evidence also suggests that disrupted mitochondrial function may also precede muscle atrophy during cancer [99], which highlights a potential role in the regulation of anabolic processes. Cancer cachexia can disrupt several processes regulating mitochondrial quality, which encompasses function, content, dynamics, and mitophagy [100]. Mitochondrial morphology and function related to ATP production and coupling efficiency are disrupted during cachexia progression [98, 101–103]. Moreover, ATP synthesis rates and electron transport chain (ETC) complex activities are reduced in cachectic muscle [101, 104–106], and tumor-derived factors can impair basal and ATP-related oxygen consumption in C_2_C_12_ myotubes [107]. Furthermore, recent genomic and metabolomic approaches have demonstrated altered carbohydrate and lipid metabolic flux coincide with mitochondrial dysfunction [108, 109]. Indices of mitochondrial quality control related to content (e.g., mitochondrial DNA expression), dynamics (e.g., Mfn1/2 and Fis1), and autophagy are disrupted throughout cachexia progression [11, 97, 98]. While these studies collectively demonstrate suppressed oxidative metabolism regulation during cancer cachexia progression, whether these alterations interact with disrupted protein turnover has not been firmly established.

### 2.6. Cancer-Induced Metabolic and Energy Stress

#### 2.6.1. AMPK Activation

AMPK activity is disrupted during cancer cachexia progression, which can alter skeletal muscle metabolism and gene expression [43]. Several preclinical models of cancer cachexia demonstrate chronically elevated muscle AMPK activity during late-stage cachexia [11, 94, 96, 110, 111], which coincides with the suppression of mTORC1 signaling and disrupted mitochondrial quality control mechanisms. While AMPK activation by systemic IL-6 also corresponds with mTORC1 suppression in tumor-bearing mice [94], AMPK inhibition could rescue IL-6-induced suppression of mTORC1 signaling in C_2_C_12_ myotubes [94]. Moreover, inhibition of myotube AMPK activity during LLC treatment improved mTORC1 signaling and protein synthesis [95]. In contrast to the acute effects of exercise on AMPK activity in healthy skeletal muscle, the chronic activation of AMPK by cachexia or IL-6 overexpression is uncoupled from mitochondrial biogenesis [11, 97]. While these studies highlight a potential role for disrupted mitochondrial function in the suppression of mTORC1 signaling during cancer cachexia, further work is needed to determine the relationship between AMPK activation in the suppression of anabolic signaling and disrupted oxidative metabolism. Given the potential role of AMPK in muscle protein synthesis and metabolic homeostasis, further work is required to determine if restoring dysregulated AMPK activity may be a potential therapeutic target for muscle wasting syndrome.

#### 2.6.2. ER Stress and UPR Activation

While muscle ER stress and UPR pathways are activated in several wasting conditions [112], their role in cancer-induced atrophy has not been widely investigated. Nonetheless, in two preclinical models of cancer cachexia, the activation of several markers of ER stress (e.g., IRE1a, XBP-1, and ATF6) was accompanied by the suppression of mTORC1 signaling, activation of protein breakdown, and dysregulated AMPK activation [113]. Moreover, tumor-derived factors within LLC conditioned media were sufficient to induce the expression of ER stress molecules and phosphorylate eIF2*α* in cultured C_2_C_12_ myotubes [113]. Interestingly, global inhibition of ER stress and UPR pathways induced muscle wasting in wild-type mice and resulted in a more pronounced cachectic muscle phenotype in tumor-bearing mice [113]. Overall, these findings demonstrate initial evidence that tumor-derived factors can induce ER stress and UPR activation in muscle; however, these pathways may also be important regulatory mechanisms to preserve muscle mass during cancer cachexia progression.

#### 2.6.3. Insulin Resistance

Related to cancer cachexia, there is evidence that mitochondrial dysfunction and excess lipid accumulation may coincide with insulin resistance [114]. Intramyocellular lipid accumulation has been observed in cachectic cancer patients [115, 116]. Moreover, the regulation of lipolysis through adipose triglyceride lipase (ATGL) and HSL activity has been negatively associated with the BMI of cachectic cancer patients [117]. The relationship between mitochondrial dysfunction and insulin resistance in many chronic disease states provides a strong rationale for impaired insulin signaling in the etiology of cancer-associated anabolic resistance in muscle.

## 3. Cancer-Induced Anabolic Resistance

Many cancer patients experience weight loss at the time of diagnosis [3], and skeletal muscle depletion is indicative of poor prognoses in many cancers [4, 118, 119]. Therefore, whether cachectic muscle retains the anabolic plasticity to treatment therapies is clinically relevant and could greatly impact a cancer patient's survival. While circadian fluctuations in protein turnover have established roles in muscle mass regulation, it is evident that protein synthesis is dynamically responsive to environmental cues. Nutrients and exercise are potent stimulators of protein synthesis in healthy skeletal muscle; however, several wasting conditions demonstrate reduced sensitivity to these anabolic stimuli [14, 120]. Given that small decrements in daily protein synthesis could significantly impact long-term muscle maintenance in wasting conditions [121], anabolic resistance could also contribute to muscle during cachexia progression. Therefore, determining the molecular mechanisms that contribute to decreased anabolic plasticity could significantly impact treatment of the cachectic cancer patient.

### 3.1. Regulation of Anabolic Resistance

#### 3.1.1. Nutrition

It has long been recognized that feeding can stimulate whole-body and skeletal muscle protein synthesis compared to the fasted state in healthy individuals [122, 123]. Similarly, consumption or infusion of essential amino acids (EAA), particularly leucine, can stimulate protein synthesis in young individuals [124–126]. The protein synthesis induction by feeding and/or EAA is associated with the activation of mTORC1 signaling [127–129]. In the presence of amino acids, the Ragulator-Rag complex targets mTORC1 to the lysosomal surface, where it can interact with and become activated by the small GTPase Rheb upon amino stimulation [130]. While the maintenance of muscle mass is critically important during aging and wasting conditions, significant progress has been made in our understanding of the anabolic response to feeding in elderly individuals [13]. Several studies have demonstrated a blunted response to lower doses of EAA in elderly individuals [131, 132], with this decreased sensitivity and responsiveness to nutrients associated with impaired mTORC1 signaling [127, 133]. However, the ability to stimulate protein synthesis remains intact when higher doses are ingested [124, 125, 134]. These findings indicate that while a threshold is needed to stimulate protein synthesis, the synthetic machinery required for protein synthesis remains intact. Related to pathological wasting conditions, muscle resistance to leucine stimulation has also been observed in severe inflammatory disorders such as sepsis and endotoxemia [135, 136]. Collectively, these studies highlight that reduced sensitivity of skeletal muscle to feeding contributes to muscle mass loss observed during aging and disease state.

#### 3.1.2. Exercise

Muscle contraction is a potent stimulator of protein synthesis in healthy skeletal muscle [14, 137] and has been shown to synergistically improve nutrient-induced stimulation of protein synthesis [138–140]. The activation of protein synthesis by resistance exercise can be observed as early as 1 h postexercise [141, 142] and can remain elevated for up to 24–48 h, dependent on the manipulation of training variables [137, 143, 144]. The cellular mechanisms associated with loading-induced protein synthesis include phosphatidic acid (PA), extracellular-related kinase 1 and 2 (ERK1/2), and mTORC1 signaling [13, 145], which can occur independent of upstream IGF-1 receptor signaling [146, 147]. Indeed, the downstream activation of P70S6K by high-force contractions has been associated with protein synthesis and muscle growth in both humans and rodents [142, 148, 149]. However, disrupted mTORC1 signaling has been implicated in the impaired anabolic response to contraction in aging and pathological conditions [142, 150, 151]. Therefore, anabolic resistance to exercise may be present in many wasting conditions.

### 3.2. Cancer-Induced Anabolic Resistance

#### 3.2.1. Nutrition

An improved understanding of the cachectic muscle response to anabolic stimuli is of great clinical importance, given that cachexia cannot be fully reversed by conventional nutritional support [2]. While emerging evidence suggests that basal muscle protein synthesis is disrupted throughout cachexia progression, few studies have examined whether nutrition and exercise can stimulate anabolic signaling in cachectic muscle. While cancer patients can still induce protein synthesis in response to protein ingestion [152–157], this anabolic response to nutrients is strongly impaired [152, 155–157]. However, a specifically formulated medical food (high protein and leucine) could overcome anabolic resistance in cancer patients with involuntary weight loss [152]. Additionally, resistance exercise could enhance the myofibrillar protein synthesis response to feeding response in men with prostate cancer on androgen deprivation [155]. These studies demonstrate that the translational machinery may be responsive to feeding, but highlight that individualized, multimodal approaches may be needed to maximally stimulate protein synthesis. Interestingly, few preclinical studies have examined the acute feeding response during cancer cachexia. However, we have demonstrated suppressed mTORC1 responsiveness to glucose administration in cachectic skeletal muscle [94]. Overall, there is clear evidence that future studies are needed to improve our understanding of the mechanisms regulating anabolic resistance to feeding, which could have significant clinical and physical ramifications in the cachectic cancer patient.

#### 3.2.2. Exercise

While the mechanisms associated with feeding-induced protein synthesis during cancer are starting to emerge, much less is known related to the acute anabolic response to contraction in cachectic muscle. We have reported that mechanoactivation of protein synthesis in stretched myotubes is disrupted by conditioned media from Lewis lung carcinoma (LLC) cells [95], suggesting that tumor-derived cachectic factors can interfere with mechanical signaling inducing protein synthesis *in vitro*. In addition, we found that severe cachexia could disrupt the metabolic and anabolic signaling response to a single bout of stimulated concentric muscle contractions [110]. However, these contractions were associated with the sustained activation of AMPK, which may be related to exacerbated metabolic stress given that cachectic muscle develops mitochondrial dysfunction [97, 102, 106]. Thus, it is imperative that future research determines the cachectic muscle's anabolic and metabolic sensitivity to different types of muscle contractions. In addition, futures studies should also examine cachectic muscle response in fasted and fed states throughout cachexia progression. Given that exercise can induce protein synthesis for many hours, the potential benefits of exercise for cachectic cancer patients will likely occur in the hours postexercise and coincide with enhanced nutritional responsiveness.

## 4. Role for Exercise Training to Impede Anabolic Resistance during Cancer Cachexia

**Figure 3:**
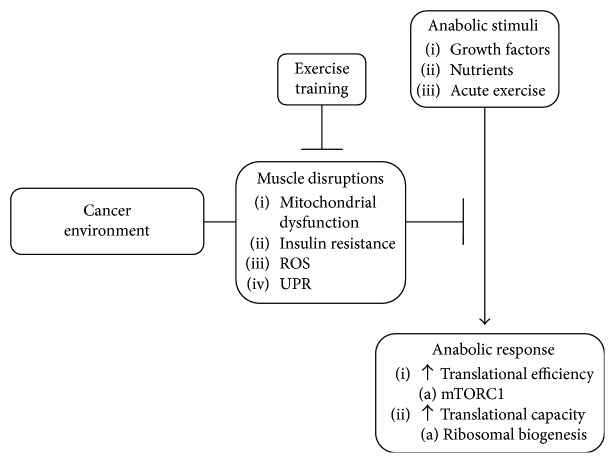
Mechanisms associated with suppressed anabolic potential during cancer cachexia. The systemic cachectic environment induces several muscle disruptions that can impede the protein synthesis induction by anabolic stimuli. Exercise training has the potential to improve muscle oxidative metabolism, which can have direct effects on protein metabolism, insulin sensitivity, and the unfolded protein response. Abbreviations: ROS, reactive oxygen species; UPR, unfolded protein response; mTORC1, mammalian target of rapamycin complex 1.

While exercise training has been discussed as a potential therapy to mitigate muscle atrophy, current understanding of the acute response and training adaptation to exercise during cancer cachexia is limited. Given that exercise involves muscle contractions that can vary in intensity and metabolic demand, the molecular responses related to growth and metabolism can differ between contraction types. In general, a single exercise bout can induce metabolic signaling pathways linked to energy production and proteostasis, whereas repeated bouts can stimulate adaptations related to oxidative metabolism and growth [158]. However, the extent to which these adaptations are related to remodeling or growth can be impacted by variables such as the exercise type, intensity and workload, and the nutritional status during the postexercise recovery [159, 160]. The following section will highlight our current understanding of exercise training to impede suppressed anabolic and metabolic plasticity during cachexia progression (Figure 3).

While the field of exercise oncology is currently limited in the number of studies that have been completed in the cachectic cancer patient, initial progress has been made in our understanding of exercise adaptations during the development and progression of cachexia in preclinical models. Treadmill exercise training alone or in combination with nutritional support can reduce tumor growth and improve muscle mass [161–163]. Moreover, we have demonstrated that treadmill training during systemic IL-6 overexpression enhanced mTORC1 signaling and mitochondrial quality control in tumor-bearing mice [94, 98], which was associated with improved insulin sensitivity [164]. Related to resistance exercise, rodent models of overload hypertrophy and stimulated eccentric contractions have demonstrated preserved muscle mass whether it was initiated prior to or at tumor-implantation [165–168]. In addition, we found that repeated bouts of stimulated eccentric contractions after the initiation of cachexia attenuated myofiber atrophy that coincided with improved oxidative capacity in tumor-bearing mice [111]. However, whether these changes were associated with improved basal protein synthesis regulation requires further investigation. Collectively, these studies demonstrate that exercise training can improve indices of muscle mass, protein turnover regulation, and oxidative metabolism throughout the progression of cancer cachexia. Additional studies are needed to determine the potential interactions between nutrients and exercise during the treatment of the cancer patient.

## 5. Conclusion and Future Research Perspectives

Muscle protein synthesis and oxidative metabolism have established roles in physical function and metabolic health. Given the significant energy requirement for protein synthesis to maintain muscle homeostasis, it is not surprising that mitochondria can regulate cellular processes related to remodeling and growth. While there is considerable evidence that protein synthesis and oxidative metabolism are disrupted throughout cachexia progression, the potential interaction between these two regulatory pathways has not been fully appreciated. Therefore, the primary objective of this review was to highlight the relationship of dysfunctional oxidative metabolism to anabolic resistance during cancer-induced muscle wasting. First, we described oxidative metabolism regulation of basal protein synthesis in skeletal muscle. Mitochondrial dysfunction has clear implications for the regulation of protein synthesis through the induction of metabolic and energetic stress pathways. While evidence exists that these pathways are altered in preclinical models of cancer cachexia, it is unclear whether this occurs in cachectic cancer patients. However, many of these studies have limitations related to sample size, patient population, and the degree and duration of the cachectic phenotype. Further research is required to establish if the mechanisms disrupting protein turnover are differentially regulated by a muscle's oxidative phenotype. Second, we assessed cancer-induced alterations to an anabolic stimulus. While not observed in all cancer patients or preclinical models, evidence exists that cachexia can disrupt the anabolic response to nutrients and exercise. However, further research is needed to clearly describe anabolic resistance throughout cachexia progression. Finally, we reviewed a role for resistance exercise to impede cancer-induced anabolic suppression and mitochondrial dysfunction. While evidence suggests that exercise maintains muscle mass in tumor-bearing mice, further investigation is warranted to determine if these improvements are related to enhanced anabolic and metabolic plasticity of cachectic skeletal muscle. Clearly defining the interactions between muscle protein synthesis, mTORC1 signaling, and oxidative metabolism will provide greater insight into the regulation of skeletal muscle wasting and systemic metabolic dysfunction during cancer cachexia progression. Additionally, understanding whether exercise training enhances skeletal muscle's sensitivity to nutrients will improve our efforts for treating the cachectic cancer patient.
